# In reply to the letter to the editor regarding “Comparison of a twin interlocking derotation and compression screw cephalomedullary nail (InterTAN) with a single screw derotation cephalomedullary nail (proximal femoral nail antirotation): a systematic review and meta-analysis for intertrochanteric fractures”

**DOI:** 10.1186/s13018-022-03244-9

**Published:** 2022-07-16

**Authors:** Leo Nherera, Paul Trueman, Alan Horner, Tracy Watson, Alan J. Johnstone

**Affiliations:** 1 Smith + Nephew Advanced Wound Management, Hull, UK; 2 Department of Orthopaedic Surgery, Saint Louis University School of Medicine, St. Louis, MO USA; 3 Orthopaedic Trauma Unit, Aberdeen Royal Infirmary, Aberdeen, UK; 4 Health Economics, Smith and Nephew Global Market Access, 5600, Clearfork Main St, Fort Worth, TX 76109 USA

## Abstract

**Background:**

Intertrochanteric hip fractures are common and devastating injuries, especially for the elderly. Surgical treatment is the optimal strategy for managing intertrochanteric fractures as it allows early rehabilitation and functional recovery. The relative effects of internal fixation strategies for intertrochanteric fracture after operation remain limited to relatively small studies which create uncertainty in attempts to establish evidence-based best practice.

**Methods:**

We conducted a systematic review and meta-analysis of randomised controlled trials (RCTs) and observational studies to assess the clinical effectiveness of two commonly used intramedullary devices: a twin-screw integrated cephalomedullary nail (InterTAN) versus a single-screw cephalomedullary nail (proximal femoral nail antirotation) in patients with intertrochanteric fractures. The following outcomes were considered: revisions, implant-related failures, non-unions, pain, Harris hip score and intra-operative outcomes. Odds ratios or mean differences with 95% confidence intervals in brackets are reported.

**Results:**

Six studies met the inclusion criteria: two randomised controlled trials and four observational studies enrolling 970 patients with a mean age of 77 years and 64% of patients being female. There was a statistically significant difference (*p* value < 0.05) for revisions OR 0.27 (0.13–0.56), implant-related failures OR 0.16 (0.09–0.27) and proportion of patients complaining of pain OR 0.50 (0.34–0.74). There was no difference in non-unions and Harris hip score (*p* value > 0.05). There was a significant difference in blood loss and fluoroscopy usage in favour of PFNA, while no difference in operating times was observed between the two devices.

**Conclusions:**

Our meta-analysis suggests that a twin-screw integrated cephalomedullary nail (InterTAN) is clinically more effective when compared to a single-screw cephalomedullary nail proximal femoral nail antirotation resulting in fewer complications, fewer revisions and fewer patients complaining of pain. No difference has been established regarding non-unions and Harris hip score. Intra-operative outcomes favour PFNA with less blood loss and fluoroscopy usage. Further studies are warranted to explore the cost-effectiveness of these and other implants in managing patients with intertrochanteric fractures.

Dear Editor

We appreciate Dr. Hu and Dr. Hui [1] for their helpful observations about our meta-analysis [2], and we are grateful to you for granting us the opportunity to respond to these observations. Our answers to the questions are as follows:

Regarding the reference by Yu et al., indeed we accept that the wrong citation was used in the manuscript; however, the data used in the meta-analysis were correct and the citation should have referred to the retrospective analysis with the following citation “Yu, W., Zhang, X., Zhu, X. et al. A retrospective analysis of the InterTan nail and proximal femoral nail anti-rotation-Asia in the treatment of unstable intertrochanteric femur fractures in the elderly. J Orthop Surg Res 11, 10 (2016). https://doi.org/10.1186/s13018-016-0344-7”.

Secondly, we would like to stress that the inclusion/exclusion table should not have included the subtrochanteric fractures. We described in the Methods section of the paper (Study selection and eligibility criteria) that we considered patients with intertrochanteric fractures. However, we note that only one study by Seyhan included subtrochanteric patients which constituted (7 of the 75 patients) 9% of the total sample size of that study and 0.72% (7 of the 970 patients) of the total meta-analysis sample size. Although we agree with the reviewer’s observation about the potential for heterogeneity, the inclusion of this study did not have any material effect on the overall conclusion of the study. Indeed, removing this study did not change the conclusions, instead the treatment effect increased in favour of InterTAN.

We were transparent from the onset that we intended to include both RCT and observational studies in the meta-analysis. We performed a subgroup analysis by study type in all our results. This was done to utilise all existing evidence as the reviewers will be aware it is difficult to conduct RCTs in medical devices. Whilst we note the observation regarding the quality checklists, we can point the reviewers to numerous meta-analyses that have been published in this and other journals which did not provide the visual risk of bias assessment and applicability tool [3–8]. It certainly can help for the visual reader; however, we do not believe that excluding the visual tool in the manuscript will automatically degrade the quality of the study.

Lastly the point on ethnic bias, we acknowledge this. External validity (“generalisability”) of findings may be lower due to differences in anatomy and patient behaviours; however, this is arguably difficult to control for in a meta-analysis. We believe this alone is unlikely to alter the conclusions of this meta-analysis. Regarding the subgroup analysis, this was by no means limited to specific studies, rather we performed subgroup analysis by study type and excluding the studies which had mixed populations which we believe was a robust way of doing it.

In conclusion, we would like to thank the reviewers for their comments on this article, and we hope our reply has helped to clarify and answer the questions they raised. Furthermore, we hope these responses are reassuring to the extent that the quality of this important work is not diminished as we believe the points raised by Drs. HU and Hui had no material impact on the overall conclusions of the paper.

## Data Availability

All data generated or analysed during this study are included in the published article.

## References

[1] Hu H, Hui Y (2022). Letter to the editor regarding “Comparison of a twin interlocking derotation and compression screw cephalomedullary nail (InterTAN) with a single screw derotation cephalomedullary nail (proximal femoral nail antirotation): a systematic review and meta-analysis for intertrochanteric fractures”. J Ortho Surg Res.

[2] Nherera L, Trueman P, Horner A (2018). Comparison of a twin interlocking derotation and compression screw cephalomedullary nail (InterTAN) with a single screw derotation cephalomedullary nail (proximal femoral nail antirotation): a systematic review and meta-analysis for intertrochanteric fractures. J Orthop Surg Res.

[3] Yu J, Zhang C, Li L, Kwong JSW, Xue L, Zeng X, Tang L, Li Y, Sunb X (2015). Internal fixation treatments for intertrochanteric fracture: a systematic review and meta-analysis of randomized evidence. Sci Rep.

[4] Liu W, Liu J, Ji G (2020). Comparison of clinical outcomes with proximal femoral nail anti-rotation versus InterTAN nail for intertrochanteric femoral fractures: a meta-analysis. J Orthop Surg Res.

[5] Hao Z, Wang X, Zhang X (2018). Comparing surgical interventions for intertrochanteric hip fracture by blood loss and operation time: a network meta-analysis. J Orthop Surg Res.

[6] Wang N, Chen Y, Ji J (2020). The relationship between serum vitamin D and fracture risk in the elderly: a meta-analysis. J Orthop Surg Res.

[7] Tian R, Zheng F, Zhao W (2020). Prevalence and influencing factors of nonunion in patients with tibial fracture: systematic review and meta-analysis. J Orthop Surg Res.

[8] Yang Z, Ni J, Long Z (2020). Is hip fracture surgery safe for patients on antiplatelet drugs and is it necessary to delay surgery? A systematic review and meta-analysis. J Orthop Surg Res.

